# Challenges experienced by induced lactation women in Malaysia: An exploratory qualitative study

**DOI:** 10.1371/journal.pone.0291758

**Published:** 2024-01-26

**Authors:** Norsyamlina Che Abdul Rahim, Zaharah Sulaiman, Tengku Alina Tengku Ismail

**Affiliations:** 1 Universiti Malaysia Kelantan, Kampus Kota, Pengkalan Chepa, Kota Bharu Kelantan, Malaysia; 2 School of Medical Sciences, Universiti Sains Malaysia, Health Campus, Kubang Kerian, Kelantan, Malaysia; Christian Medical College, INDIA

## Abstract

**Background:**

Induction of lactation occurs when breast milk is produced in a human (woman), without going through the process of pregnancy and delivery. Efforts to produce milk by women who had never been pregnant and give birth are not easy. According to the many who have attempted it, it is far more arduous a task than initiating postpartum lactation, but it is possible and worth the effort.

**Research aim:**

This study aimed to explore and understand the challenges for women during induced lactation in Malaysia.

**Methods:**

This qualitative study was done in 2017 and utilized in-depth interview among women who induced lactation in five states based on five regions in Malaysia [Central Region (Selangor), Northern Region (Penang), Southern Region (Johor), East Coast Region (Kelantan), Malaysia Borneo (Sabah)]. All interviews were consented, audio-recorded then transcribed verbatim, followed by identification of main themes.

**Results:**

Data saturation was achieved after interviewing a total of 23 induced lactation women. Data synthesized using thematic analysis revealed six themes describing the main challenges during the induction process were (1) health condition, (2) work commitment, (3) overexertion, (4) not eligible to take leave, (5) inability to follow the treatment plan, and (6) difficulty attaching the adopted baby to initiate breastfeeding during induced lactation process.

**Conclusions:**

The challenges faced by women during the process of induced lactation were complex and the induced lactation process requires considerable dedication and determination. The findings of this research will help other women and their spouses/partners comprehend the challenges, obstacles, and support that are needed along the induced lactation process. The Government and other stakeholders have roles in more efforts and programs to help these mothers breastfeed their adoptive child and make them feel more accomplished as women and mothers.

## Introduction

Induced lactation is a method of stimulating the production of breast milk in women who have not gone through the process of pregnancy [1]. Past research in Western countries explored the breastfeeding of adopted children, as well as the perceptions, achievements, and challenges of this process [2–5]. Advances in medical science and technology, especially induced lactation, have given women the ability to breastfeed children to whom they did not give birth [6]. Many women claimed that the choice to breastfeed their adopted child makes them feel more accomplished as women and mothers. Such an approach has been found to create stronger maternal-infant bonding between the adopted child and parent [7]. Women who have never had children or breastfed have the potential to produce milk much as a woman who has been pregnant and breastfed [8]. However, various challenges need to be addressed, and plans need to be made to ensure that breastfeeding is achieved.

In Malaysia, the most recent data on estimated exclusive breastfeeding in the first six months of life was 40.3% [9]. However, there is no information on the prevalence of induced lactation in Malaysia. Lactation consultants and medical professionals provide services and facilities for inducing lactation in Malaysia, which are widely available. Breastfeeding Support Centre (Susuibu.com) reported only eight out of 17 of women (47.2%) who sought help with induced lactation at their facility succeeded [10].

Others, meanwhile, have not been able to give their adopted children breast milk. This private facility focuses on providing high-quality breastfeeding consultation services, as well as teaching and training to healthcare providers and the general public. According to a Malaysia National Lactation Centre (NLC) survey in year 2019 [11], 85.0% (n = 17) of women who have never been pregnant were able to breastfeed after breastfeeding consultation services. However, only 30.0% (n = 5) of breastfeeding mothers achieved *mahram* status throughout the national breastfeeding program. The term *mahram* is used to denote a level of relationship between close family members, i.e., those with whom the hijab does not have to be observed [12]. After the mother breastfeeds the child (i.e., gives them a full breast milk meal) at least five times before the child reaches the age of two, an adoptive mother and her adopted child are deemed to have developed a *mahram* relationship [13]. This situation indicates that the process requires effort and patience to overcome the challenge involved in the process. Mental strength, physical and spiritual readiness are also important because the successful implementation of the breastfeeding process will face many challenges.

Awareness among adoptive mothers and their determination to breastfeed are important. In addition to the technical aspects, emotional and spiritual support are also important for successful breastfeeding. However, there are many factors to be considered in induced lactation programmes, due to adoptive mothers’ varying religious and ethnic backgrounds, health status, financial and environmental challenges. Previous studies conducted in Western countries have commonly explored the breastfeeding of adopted children, as well as the experiences, successes, and challenges of this process. Despite the abundance of research in the field, there is a lack of clarity due to a paucity of studies on induced lactation practise in Malaysia.

The process of induced lactation in Malaysia can be further explored by identifying and understanding women’s challenges. To date, there is no national data available for induced lactation, although most hospitals and health clinics in Malaysia have provided induced lactation services. There is a lack of clarification due to limited research on induced lactation practices in Malaysia. This study’s findings could help other women and their spouses/partners comprehend the challenges, obstacles, and support that are needed along the induced lactation process. In Malaysia, induced lactation is becoming more widespread as adoption becomes more common, with 1,424 adopted children in 2018 [14] and 1,799 adopted children in 2019 [15]. The government and other stakeholders should undertake more efforts and programs to help these mothers breastfeed their adopted child and make them feel more accomplished as women and mothers. Intense passion, deep desire, patience, and most importantly, sincere intentions, are the root of success in the process of raising an adoptive child [16]. Therefore, this study aimed to explore and understand the challenges for women during induced lactation in Malaysia.

## Materials and methods

### Study design and setting

This exploratory study using a purposive sampling method, conducted from June 2017 to November 2017 involved five regions in Malaysia representing the East Region, North Region, South Region, Central Region, and Borneo. A state was selected from each region randomly to ensure various experienced throughout the country are captured. The chosen states were Kelantan, Penang, Johore, Selangor, and Sabah. The study adopted an exploratory qualitative approach to establish a comprehensive insight into the women’s experiences throughout the journey of induced lactation. The design allows exploring respondents’ feelings, behavior, thoughts, insight, and action [17]. The detailed methodology of this study was described and published by Che Abdul Rahim et al. [18]. This study was authorized by the Human Research Ethics Committee University Sains Malaysia with the code project USM/JEPeM/14044139 and the Medical Research Ethics Committee (MREC), Ministry of Health of Malaysia (MOH) and was registered under NMRR-15-1600-26147 (IIR). Written informed consent was secured from study respondents after explaining the study’s objective and purpose to each study respondents. The respondents were also assured about the confidentiality of the data.

### Sample

The selection of women who was undergoing induced lactation procedure (completed or still under treatment) was obtained from the practitioners who managed case of induced lactation at lactation centers, hospitals, and health clinics. The practitioners provided one to ten names of women who were their clients based on the inclusion and exclusion criteria: woman, has never been pregnant, do not have a biological child, the adopted child must not be more than two years according to the lunar month calculation during data collection, no previous breastfeeding experience, and undergoing induced lactation procedure (completed or still under treatment). The sample size is estimated based on the saturation concept for qualitative research [19]. After interviewing a total of 23 induced lactation women, data saturation was obtained. The saturation occurs when new data collection has no longer contributes to further information on the issues under investigation.

### Data collection

In-depth interviews were conducted using an interview guide exploring respondents’ experiences and their perspectives on induced lactation process. The interviews were conducted at home settings, and some were conducted in restaurants as per respondent’s request. Interviews were performed in the Malay language because most of the respondents were Malays, and they understood and preferred the language. The saturation occurs when new data collection has no longer contributes to further information on the issues under investigation. The respondents provided written consent, and brief socio-demographic information prior to the interview sessions. Confidentiality and anonymity of ethics were maintained. All interviews were conducted for approximately 45 to 90 minutes each session.

### Data analysis

The interviews were recorded with some notetaking by the researcher. The Malay language audio files were transcribed verbatim. These interview transcripts were then coded and managed using ATLAS.ti Version 8 software. Thematic analysis was chosen as the method to analyze the data from the interviews. The analysis represents a systematic framework to code qualitative data to recognize patterns across the data [20]. Six discrete steps are involved in thematic analysis such are (1) become familiar with the data, (2) generate initial codes, (3) search for themes, (4) review themes, (5) define themes, and (6) write-up [21].

In order to enhance the credibility of the study, triangulation from different sources has been applied by researchers. To assure information quality, the interview was supplemented with secondary data from home and hospital-based information sheets. Any discrepancies between the data presented on the information sheet and the data acquired during the interview were verified. Next, the member checking process was done by phone calls, and the polished content of the transcripts and the quotations cited were read to the respondents. Rich, thick description was utilized to provide various viewpoints on a theme, making the data more genuine and realistic. The study procedures were clearly documented in order to allow reproducibility [22].

The transcription notes were checked repeatedly with the audio files to minimize obvious errors. Furthermore, triangulation of data includes conducting a pilot study, multiple case studies, triangulation between case study interview data and respondent’s verification of interview data. Rigor was tested in this study to assess research quality and ensure that the data obtained are as relevant and trustworthy as feasible [23]. Rigor was evaluated by means of dependability, credibility, confirmability, and transferability.

## Results

### Respondent characteristics

Out of 23 women undergoing induced lactation procedures, six respondents were housewives (26.0%) and 17 respondents worked in the government or private multi-sectors (74.0%). The respondents aged 26 to 40 years. Two respondents had two adopted children (one respondent had a twin as adopted children). The others only adopted one child. In total, 25 children were adopted from 23 women; 13 children were boys (52.0%), and 12 children were girls (48.0%). The adopted children’s ages ranged from 20 days to 24 months at the time of the interview. Respondents in this study were mostly ethnic Malay Muslims.

The mean duration of marriage among the adoptive couples was nine years (with marriage duration ranging from four to 15 years). Seven women were married for more than ten years, 15 women were married for less than ten years, and one woman was single and not married. Those married for more than ten years were all between the ages of 32 and 40. Out of the 15 women married for less than ten years, three were between 26 and 38. All were in their reproductive age, which is 26 to 40 years old. Their background characteristics are shown in Table 1.

**Table 1:** Descriptive characteristics of respondents.

Respondents	Age (year)	Occupation	Duration of Marriage (year)	Adopted child	
				Sex	Age of child during interview (month)
R1	37	Housewife	8	Girl	5
R2	32	Housewife	11	Girl	8
R3	38	Teacher	12	Boy	12
R4	38	Lecturer	NA	Girl	12
R5	38	Liaison officer	9	Girl	19
R6	33	Lecturer	8	Boy	24
R7	27	Housewife	4	2 (Boy & Girl)	19
R8	34	Housewife	5	Boy	2
R9	36	Teacher	12	Boy	3
R10	34	Teacher	10	Boy	12
R11	40	Librarian	13	Girl	24
R12	38	Nurse	12	Girl	8
R13	34	Industry officer	9	Boy	4
R14	31	Housewife	7	Girl	4
R15	29	Lecturer	4	Girl	4
R16	40	Administrative assistant	13	Girl	24
R17	30	Accountant	9	Girl	20 days
R18	34	Safety guard	7	Boy	20 days
R19	26	Assistant pharmacist	5	Boy	1
R20	36	Medical laboratory technologist	9	Boy	20
R21	35	Nurse	10	Girl	2
R22	37	Teacher	10	Boy	1
R23	36	Housewife	15	2 Boys (Twin)	12

### The challenges for women during the process of induced lactation

Based on the interviews with 23 respondents, only 17 women managed to produce milk and breastfeed their adopted babies, while six women were still in the process of induced lactation when the interview was conducted. However, the length of the induced lactation process starting until successful breastfeeding was between three weeks until 22 months. This indicates that various challenges were faced during the process of induced lactation. There were six main themes that explained the challenges experienced by the women, which were: (1) health condition; (2) work commitment; (3) overexertion; (4) not eligible to take leave; (5) inability to follow the treatment plan; and (6) difficulty attaching the adopted baby to initiate breastfeeding. Table 2 presents the themes, sub-themes, and codes representing the challenges experienced by women who induced lactation.

**Table 2:** Themes sub-themes and codes of the challenges of women who induced lactation.

Code	Sub-theme	Main-theme
• Polycystic ovary syndrome. • Kidney failure. • Hepatitis B. • Ovary cyst. • Cancer. • Heart problem. • Declining health status.	• Health issues specific to women health. • Chronic health condition.	(1) Health condition.
• No break times. • Irregular work schedule. • Tight schedule at workplace. • Overtime. • Night shifts.	• Time constraint. • High responsibility at the workplace. • Nature of working/shift work.	(2) Work commitment.
• Not enough sleep. • Being forced to stay up at night. • Need to babysit the small child. • Being force to maintain breastmilk production. • Lack of confident. • Upset due to the fact they could not produce enough milk to meet demand.	• Worried. • Fatigue. • Stress.	(3) Overexertion.
• National policies on maternity leaves not provided for induced lactation practices. • Insufficient time to start preparations for induced lactation. • Could not apply for leave as early as the baby was received unexpectedly. • Cannot take unpaid leave due to an immense financial obligation.	• No maternity leaves. • Have a lot of commitment at the workplace. • Employers are not allowed to take leave.	(4) Not eligible to take leave.
• Worry about the baby not getting enough breast milk. • Less effort to do breast stimulation. • No urgency to hasten the process of producing milk. • Faced some problems during the breastfeeding (i.e., cracked nipples, nipple blebs, or milk blisters and stiffened areas of the breast due to the stimulation).	• Inconsistent stimulation of the breasts. • Doubt about milk supply. • Physical difficulties • Nipple condition.	(5) Inability to follow the treatment plan.
• Baby has been introduced to bottle feeding from the start. • Age of baby at the time when the induced lactation process started. • Baby introduced artificial teat early. • Baby difficult to suckle on the breast. • Growing child did not want to be on direct feeding.	• No previous breastfeeding experiences. • Baby refuses to get the breast. • Inadequate attachment of the baby to the breast. Inadequate knowledge and skill.	(6) Difficulty attaching the adopted baby to initiate breastfeeding.

#### Health condition

Respondents need to follow certain induced lactation protocols and specific techniques consistently to stimulate milk production without pregnancy. However, the process of successful induction is not as easy as they thought. One of the factors that could interrupt the process of induction was the adoptive mother’s health condition. Most of the women interviewed said that they had a health issue that affected the process of induction. This health condition necessitates that woman take medication and engage in specific treatments, which makes it difficult to continue taking medication for the process of induced lactation. Among the stated diseases were health issues specific to women health such as polycystic ovary syndrome, ovarian cyst, chronic health condition, Hepatitis B, cancer and heart problem, as mentioned below:

“I need to drink a lot of water to make sure the milk supply is always available. If I drink a lot of water, I will have difficulty breathing due to my liver disease. I am on treatment, and I need to take 20 pills every day for my heart and liver problems.” (R1)

Another woman remarked,

“I am worried that I cannot breastfeed the baby as I am suffering from cancer. I do not know how to breastfeed the baby. I am taking various drugs, and the latest one is to strengthen the bone after the operation.” (R14)

However, some of the respondents did not have a medical condition/fertility problem diagnosed by a doctor. They used the terms ‘no health problem’, ‘no fortune yet’, ‘doing IVF and IUI’ and ‘fertility treatment’:

“I am not suffering from fertility problems and other illnesses. If I want to get pregnant, I need to do an IVF (in vitro fertilization).” (R19)

Impaired health conditions also disrupted milk production. This was acknowledged by one of the respondents:

“I tried to stimulate the breast for two to three weeks, then my nipple starts exuding fluid. After I had a fever, the milk stops to come out, even with pumping.” (R13)

#### Work commitment

From a theoretical point of view, the process might look simple and easy to be done. However, not all respondents that start the induced lactation process can produce milk at the final stage. Constraint from a career perspective also affects the effort to breastfeed the adopted child.

One of the respondents stated that her break time and irregular work schedules left her with little time to do the milking:

“Uncertain break time and we need to do rotation. After me, other people get his break at 12, and some get their break at 10. It is difficult for me to go out to pump my breast since there is no time and the break is not that long.” (R18)

The time constraints in the workplace make it impossible for most respondents to do breast pumping at work. The woman stated that the difficulty to do breast pumping at workplaces were the factors that drove them to stop doing so, as mentioned by one of the participants:

“If we are not putting enough efforts, the results will not be there. I have to admit that I am not really committed because of workload and restricted time.” (R11)

Some respondents had produced milk before returning to work, but the milk supply was reduced when they returned to work due to a tight work schedule:

“I need to consume medicine to produce milk. Somehow due to a packed schedule and inconsistent time of pumping, the milk supply is reduced, and at last, it stopped.” (R10)

Work routines that require overtime and night shifts were the obstacles faced by one of the respondents to continue the breast pumping schedule:

“Because of work and unpredictable overtime shifts, I do not have enough time to pump my breast. I work as a security guard and work in irregular shifts. At times I need to replace my colleague and do overtime; that is why I do not have time to pump.” (R18)

#### Overexertion

The respondents experienced five types of situations during breastfeeding, i.e., *‘*not enough sleep’, ‘worried’, ‘fatigue’, ‘stress’, and ‘lack of confidence’. One of the respondents stated that she could not get enough sleep from babysitting a small child:

“It is incredibly challenging. When I first had the baby, I need to handle the feeding and the small baby. I could not get enough sleep, as the baby also stayed up at night.” (R3)

Being forced to stay up at night in addition to the commitment at work caused one of the respondents to face the stress that interferes with the breastfeeding process that has occurred before:

“I am really tired, and my eyes were swollen from staying up at night. The baby becomes cranky since he is always bloated. His stomach is bloated from cow’s milk intolerance. I am feeling so frustrated because of work stress. If not because of the stress, I might be able to breastfeed him till now. But my work is stressful.” (R12)

One of the respondents described her lack of confidence and inability to take care of her child alone, making her unable to properly stimulate milk production. She stated that she could not follow the schedule of breast pumping due to time constraints, plus the need to babysit the small child. She expressed the feeling as below:

"I am not confident. After all, consistency is the key. The doctor advised us to do pumping every two hours, and if we have free time, we should make double pumping with a 10-minute interval. After that, we will pump it in half an hour. We also must relax, keep calm, and avoid stress, but the problem is whenever I start to pump, the kid starts to cry. Maybe there is a mistake somewhere, but I have to be strong and keep trying." (R7)

Feeling worried, stressed, exhausted with childcare, and consistency in breast pumping caused them to think that the process of induced lactation is particularly challenging. They expressed their feeling as below:

“My child often cries. It is hard, and it is really depressing. But after that, my milk starts to increase in amount after two weeks of consuming the medicine. I feel relieved, though." (R16)

“Dr A said that oxytocin is high at midnight and in the morning. If we do pump during that time, we will produce milk faster. But I am really tired during that time, and I cannot do it. I am working from 8.00 am to 5.00 pm, and my husband furthers his study. He is doing industrial practical, so he is unable to be at home at 5.00 pm. He came home around 5.30 pm. So, I must pick up my child from the babysitter at 6.00 pm and arrive home during Maghrib. To complete eight to 12 times of pumping is impossible. I am really tired.” (R20)

#### Not eligible to take leave

In Malaysia, the national policies on maternity leave also pose a threat to induced lactation practices. About half of the workforce is women, and generally, they are entitled to eight to 12 weeks (60 to 90 days) of paid maternity leave depending on the employer’s consent. However, this privilege is not given to a mother who wishes to breastfeed her adopted child. The mother had to take leave on her initiative if she wishes to undergo the induced lactation program. These circumstances forced the mother to give up so easily because of the burden in the workplace, and at the same time, she had to prepare to breastfeed her adopted child in a limited time.

Some of the respondents mentioned that no leave for babysitting was one of the reasons why they do not have time to prepare for implementing the process of induced lactation:

“The doctor advised me to take a leave for two weeks, if possible, if not, just take a leave for one week. I do not know how to explain to the employer the reason for leave, so I cancelled it.” (R11)

One of the respondents stated that the employer did not provide maternity leave, so she had to use the cumulative leave:

“Since I already took many leaves before, my employer did not allow me to take another leave.” (R18)

Some of the respondents cannot take leave to make some preparations because of high working commitment and financial problem:

“My main constraint is that I do not have three months of maternity leave. I do not ask for leave since it is unpaid leave. I have so much commitment each month, and I have to pay a lot of bills, so I do not apply for a leave.” (R4)

#### Inability to follow the treatment plan

Inadequate supply of milk is one of the most common reasons they stop breastfeeding their adopted child, because of the inability to follow the treatment plan.

Most respondents acknowledged that the most critical challenge was to be consistent in stimulating their breast milk production:

“The biggest challenge is us. We must be strong to follow the schedule of pumping, added with some prayers, but it is only hard when you are not committed. I think this is my fault, not doing the pumping. I do not commit to [pumping] regardless [of] all other efforts.” (R9)

Meanwhile, a participant informed that she had no urgency to hasten the process of producing milk and subsequently to breastfeed the adopted child, as it still has a long time (two years):

“I do not feel the urge to pump. Sometimes, I missed it. Sometimes, I skip it one whole day, and only pump at night. It is troublesome to pump. I still have two years to do it.” (R4)

Inadequate supply of milk was usually seen as an impression of the "real" condition of milk supply, and this suggests that there are other factors that cause respondents to have doubts about their milk supply. One of them expressed the feeling as below:

*“*I take the baby when I am already expressing milk. I collect them but only get half an ounce, twice. I get another half an ounce when the baby is a month and a half year old. There is not enough milk for the baby. Feeling broken after trying for three weeks, I only get one drop of fluid. Now I get more than that, but still not much.” (R13)

Some respondents were worried about the quantity of milk produced, as the baby was on direct breastfeeding, but only a little amount of milk came out when being expressed:

“I already took medicine, and I am producing milk right now. Still, I am in doubt whether the milk is enough for the baby or not. I feel worried. I am confused because I start to produce milk, but only a little milk comes out when I try to do the milking. The milk does come out, but extraordinarily little. But I do not know what to do. I am not sure if I can give him my breastmilk only or not. I am not confident.” (R18)

Some of the problems faced during the breastfeeding include cracked nipples, nipple blebs or milk blisters, and stiffened areas of the breast due to the stimulation, as recounted by one of the participants:

"The first thing I remember is that the doctor told me when I have the baby to let him latch on the breast. At first, of course, I did not know how to [do it]. I feel ticklish. After trying a few times, my nipples crack. It hurts so much. At that time, I want to give up. But when I see the doctor, the doctor tells me to continue when the nipples heal. I try to use the correct way of feeding, and all is okay, Alhamdulillah.” (R4)

#### Difficulty attaching the adopted baby to initiate breastfeeding

Starting breastfeeding was challenging for a woman who has no experience in breastfeeding, not to mention the child was not her biological child. A participant has recounted the challenges:

“I have no experience and no one to teach me. It differs from the biological mom, who nurses, or midwives will attend. I do not know where to learn since I have nobody to teach me. So, I learn by myself.” (R4)

Some of the respondents also stated that the baby might have learned to suckle on an artificial teat and find it difficult to suckle on the breast:

"When I took the baby, he was not a fussy baby. He was fine with a bottle. His biological mother was breastfeeding him. I want to do so at first, but I am not producing milk yet, so I feed him through a bottle. Soon after that, I realized that he became accustomed to the bottle and refused to breastfeed.” (R20)

Some respondents stated that the growing child did not want to be on direct feeding. This condition caused inadequate attachment of the baby to the breast. The babies also need a larger quantity of milk, and respondents’ body cannot meet the demand:

“The problem is he eats too much since he’s grown up. He drinks four ounces of milk all at once, and he becomes full after that. I want to restart the breastfeeding as I am not feeling satisfied. He does not want to suck my breast.” (R11)

## Discussion

Our study respondents faced six types of challenges namely (1) health condition, (2) work commitment, (3) overexertion, (4) not eligible to take leave, (5) inability to follow the treatment plan, and (6) difficulty attaching the adopted baby to initiate breastfeeding during induced lactation process This present study is consistent with the previous findings, which indicate that adoptive mothers may face specific challenges in breastfeeding [5,16,24,25]. Lommen and Brown [16] also concluded that adoptive mothers encounter feelings of rejection, anger, stress, and failure during the induced lactation process. However, with the support from breastfeeding programs and their families, they could breastfeed their babies successfully.

Efforts to produce milk by respondents who have never been pregnant and give birth are not easy. They need to consider several factors before enrolling induced lactation program since everyone’s needs are different. The findings were consistent with those found in the literature, where the implementation of breastfeeding is a complex issue, and the induced lactation process requires considerable dedication and determination [2–5,7,26]. Previous studies revealed that biophysical factors (pain, nipple injury, and insufficient milk), psychosocial factors (maternal motivation and confidence, breastfeeding knowledge, family support, and breastfeeding intentions), and socio-demographic factors (household income, maternal education level, and return to work) are significant challenges during the induced lactation process [5,8,24,25,27–29].

Most respondents emphasized that the main challenges for attempting induced lactation were health conditions. These findings are in line with the study by Siti Mariam [30], who stated that breastfeeding’s main challenge is health problems. Women with health problems faced difficulty with producing milk through induced lactation compared to healthy women. In the pharmacological method, the women took galactagogues or hormonal treatments (estrogen and progesterone). Hormonal treatments create a hormonal state similar to the condition that occurs during pregnancy. The health risks associated with using the galactagogues or hormonal treatments include weight gain, headache, gastric problem, depression, and irregular periods. However, according to Zaharah and Tengku Alina [31], among the categories of women who can undergo the process of induced lactation are women without ovaries, women without wombs, women who had not been pregnant or given birth, women who had reached menopause, women whose pregnancy ended in miscarriages, and pregnant women whose infant died in the womb or after birth. Anatomical factors, such as having an ovary and womb, or the actual birth of a baby, are not factors that will affect milk production [1].

The majority of women in this study claimed that their work commitments hinder them from being disciplined in the ongoing lactation process. Studies in breastfeeding practices among working women have revealed that they neglect their responsibility to provide their baby with breast milk needs [32]. This is in line with Ramiro *et al*. [29], who stated that the main reasons for breastfeeding cessation are perceived milk insufficiency and returning to work [33]. However, there are no data available regarding the prevalence of induced lactation in Malaysia [34]. The main challenges in induced lactation among Malaysian women were non-conducive work environments and career setbacks [30].

According to Da Rocha *et al*. [35], the process required an effort that could be stressful but rewarding when the mother could breastfeed her baby. The most challenging part of our respondents’ process was the round-the-clock stimulation of the breasts that caused overexertion. However, it should be indicated that induced lactation can also be attained by putting the baby to the breast after the baby arrives [29]. This option should be kept in mind to avoid the onerous process that our study respondents went through; thus, reducing stress.

Working women in this study reported that they were struggling to get an adequate leave of absence from work of at least two weeks to prepare for the process of induced lactation. There is no special maternity leave for mothers who do not give birth to a baby. In Malaysia, national policies on maternity leave also affect induced lactation practices. According to the Service Circular Letter Number 15 the Year 2007 and Service Circular Letter Number 14 the Year 2007 [36], the Convenience Leave to Take Care of a Child without any payment had been allocated to female employees to take care of and breastfeed their babies, and similarly for female employees who have stepchildren under their care or adopted children who had been adopted legally. The period is limited to one year during the time of service or before the child reaches the age of five years old, depending on which comes first. However, high commitments do not allow women to take leave without any payment or salary. Ashmika [37] stated that the reasons for the termination of breastfeeding by women who breastfeed are due to return to work at the end of the maternity leave. Her survey showed that 32.5% of survey respondents reported that they had to stop breastfeeding because they wanted to resume their careers. The findings of this study were also supported by Hapsah *et al*. [38] when they stated that one of the main reasons’ women did not continue breastfeeding exclusively was work.

Most of the women acknowledged that the most critical challenge was to be consistent in stimulating their breast milk due to their inability to follow the treatment plan. Theoretically, the basic concept and procedures for inducing lactation seemed straightforward, but almost all women face severe difficulties in practice. Zilal and Farahwahida [6] found that not all women who attempted induced lactation could produce milk, often due to a poor understanding of the concept. In many cases, the mother had limited exposure to the knowledge of induced lactation protocols and procedures, which frequently led to further problems after adoption. Researchers have identified that induced lactation’s success factors were the ability to follow the treatment plan, including frequent stimulation of breasts and sucking by the baby [24]. The inability to follow the treatment plan may cause the adoptive breastfeeding process to fail. Incorporating treatment plans in the daily schedule is essential for the study’s respondents as the goal of adoptive breastfeeding. Therefore, the respondents need to arrange and manage their time wisely to meet lactation’s objective within a specified period. Comparing the results with Zilal *et al*. [39], time management involved three things, i.e., taking unpaid leave, the time to express milk, and giving breast milk to the baby. Kinga *et al*. [5] also reported similar observations in their study, where adoptive breastfeeding achievement reflects careful planning by the adoptive mother beginning in the prenatal period, her active role during the adopted baby’s hospital stays, and support health care personnel and family members.

The most important reason for the early introduction of infant formula was the mothers’ perception of insufficient breast milk production [40]. Based on the results, many adoptive mothers remained unfamiliar with the techniques and preparation required to induce lactation effectively despite having received guidance from the practitioner. This is due to married couples who wish to induce lactation being exposed to content about such services on social media platforms, such as Facebook, blogs, websites, and family magazines. The majority of women stated that the difficulty in getting the adopted baby to initiate breastfeeding at the beginning of the adoption led to the women being depressed and opting to use infant formula and making it more difficult to produce milk. Poor breastfeeding techniques were reported among the individual factors associated with unsuccessful breastfeeding, indicating that adequate breastfeeding support, including evaluating latching, position, and feeding at the breast, could prevent nipple cracks, and mastitis [41].

The challenges of breastfeeding itself that our study respondents faced were similar to those reported for gestating mothers: breast health and milk supply [42]. Similar to a previous study by Hackman *et al*. [43], we showed that participants who breastfed for the first time often had more difficulty initiating breastfeeding than those who had breastfed previously. Concerning their doubts about milk supply, similar to other findings [25,35,44], the type of feeding (i.e., exclusive breastfeeding, predominant breastfeeding, mixed feeding, or bottle feeding), and the inability to exclusively breastfeed a child, did not adversely affect the participants. This finding coincides with their belief that the mother-child relationship’s benefit was significant regardless of how much milk was produced.

To gain knowledge, every woman is encouraged to visit a certified medical practitioner, lactation consultant, or lactation counselor face-to-face to receive a comprehensive overview of the lactation-inducing process [45]. Medical practitioners can help the mothers by providing knowledge on the effective techniques to fulfill this unique desire [2] successfully. Direct consultation with experts offers women a much clearer view of the available options and a greater understanding of the best practices for adoptive breastfeeding. All respondents’ efforts and commitment to gathering information designate the importance of knowledge for adoptive breastfeeding to succeed [34]. The ability to provide breast milk to their adopted child is why these women successfully breastfeed.

Finally, the respondents’ motivation for breastfeeding was to improve their sense of closeness to their infants, and they reported that their expectations had been fulfilled. In future studies, researchers should compare the feeling of closeness to one’s infant between non-gestating mothers who breastfeed their infants and those who do not.

## Strength and limitation of the study

This study’s main strength is that we have conducted this study across Malaysia, which represents the five regions. Women in the current sample reported having no children of their own; however, as questions primarily focused on societal attitudes towards adoptive breastfeeding, the nulliparous sample offered unique perspectives related to perceived social norms of breastfeeding. This study also used primary data that should consider strength. However, because the information was obtained retrospectively, one to two years after the induced lactation procedure was completed, there may be recall bias among the respondents. As a result, we cannot rule out recollection bias in the interview responses. In this sense, the reported adoptive breastfeeding rates may have exceeded the real rates. The fact that all of the women in this study who underwent induced lactation were Muslims limits the findings’ applicability to other religious groups. The women who underwent induced lactation in this study were ethnic Malay Muslims. As a result, comparisons to other ethnic groups were impossible. This was owing to the difficulties in recruiting women from various ethnic groups who had undergone induced lactation for the study. Because the data was collected in 2017, induced lactation may be more widespread in Malaysia currently, and thoughts against it may have shifted in the previous five years.

## Conclusion

In conclusion, women’s health condition, work commitment, overexertion, not eligible to take leave, inability to follow the treatment plan, and difficulty in getting the adopted baby to initiate breastfeeding were the challenges faced among women during the process of induced lactation in this study. However, efforts to breastfeed the adoptive child through the lactation induction program are not impossible. Adoptive mothers need to understand how this process works, follow the appropriate protocols, and implement breast stimulation with discipline. The cooperation of the adoptive mother and baby is also crucial during the breastfeeding process. Researchers equate the lactation process with everyday life where positive attitudes are followed by a strong desire to succeed. Our findings can inform clinicians and other researchers about the experiences of women during induced lactation. Furthermore, we hope that health professionals and lactation support providers will consider it when providing care during these procedures.

## Abbreviations

IVF: In Vitro Fertilization
IUI: Intrauterine insemination
IBCLC: International Board of Certified Lactation Consultants
